# Techno-uncertainty and organizational counterproductive work behavior among Generation X employees in a South African state-owned enterprise: the moderating role of gender

**DOI:** 10.3389/fpsyg.2025.1656654

**Published:** 2026-04-24

**Authors:** Crispen Chipunza, Lovemore T. Chipunza, Samson Adewumi

**Affiliations:** 1 Department of Business Management, Central University of Technology, Free State, South Africa; 2 Rural Electrification Agency, Mutare, Zimbabwe; 3 Graduate School of Business Leadership, University of South Africa, Midrand, South Africa

**Keywords:** counterproductive work behavior, state-owned enterprises, techno-uncertainty, gender, technology

## Abstract

The increasing adoption of digital technologies in organizations continues to raise concerns about their impact on employee behavior. Although prior studies have examined technostress as a composite construct, limited attention has been given to how its individual dimensions influence negative workplace behaviors across different demographic groups. The purpose of this study was therefore to examine techno-uncertainty and its effect on organizational counterproductive work behavior (CWB) among Generation X employees, and whether gender moderates this relationship. Quantitative data were collected from 230 full-time Generation X employees working at a South African State-owned Enterprise. The findings revealed a statistically significant positive effect of techno-uncertainty on organizational counterproductive work behavior. Gender moderates the relationship between techno-uncertainty and organizational counterproductive work behavior. The study contributes to the literature on the consequences of technological uncertainty in digitally transforming workplaces, particularly among Generation X employees, while also demonstrating the importance of examining specific technostress dimensions in isolation. The findings provide practical relevance for organizations in terms of designing inclusive, generation and gender-sensitive digital transformation strategies in African workplace contexts.

## 1 Introduction

The concept of techno-uncertainty pertains to the anxiety and confusion caused by the rapid introduction and use of technology, as well as its impact on an individual’s work (Pflügner et al., 2021). For Generation X employees, born between 1965 and 1980, known as “digital immigrants” who adopted technology as adults (Calvo-Porral and Pesqueira-Sanchez, 2020), and often characterized by a techno-averse attitude or skepticism toward technology (Prensky, 2001), as well as being individualistic, ambitious, and frequently workaholic to an addictive degree (Gurau, 2012), adapting to these swift technological changes can be challenging (Vogels et al., 2020). Research suggests that techno-related uncertainty may lead to counterproductive work behaviors—deliberate actions that harm organizations, such as fraud and theft (Spector and Fox, 2005). However, there is limited evidence regarding how gender influences this relationship. Gender-specific responses could help explain how males and females react differently to the same stimuli (technology), potentially affecting their susceptibility to counterproductive work behaviors. Such insights may help State-owned Enterprises (SOEs) develop appropriate and inclusive management strategies. In South Africa, there has been a gradual adoption of technology, with notable increases in automation and Artificial Intelligence (AI)-driven processes and solutions (Jaiswar, 2024). While the rates of adoption vary by sector, State-Owned Enterprises (SOEs) have navigated rapid technological changes amid increasing pressure to remain viable and competitive (Malope et al., 2021). The SOEs are known for employing a large number of Generation X employees, who are in senior or middle management positions and serve as a bridge between them and younger employees (Purwadi et al., 2022). These employees face unique pressures as they navigate rapidly evolving technologies (Sansovini and Magida, 2025). Retaining these employees is crucial for maintaining stability, as they possess institutional knowledge and experience. Despite leading in technological adoption, reports of poor service delivery, corruption, and bribery (counterproductive work behaviors) among SOE employees are common (Chitiga et al., 2022). In addition, audit reports on several SOEs in the country have highlighted cases where employees are feeling overwhelmed by the introduction of digital systems, unclear Information Technology (IT) guidelines, and continuous software upgrades (Auditor-General of South Africa [AGSA], 2025). Similarly, an operational report from one SOE in the country noted that almost 60% of Generation X employees rated their familiarity with new enterprise systems as low, citing a lack of training on system changes (BusinessTech, 2025). These facts indicate the real-world context of techno-uncertainty—the unpredictable levels of stress and anxiety associated with technological changes in the workplace.

Additionally, gender mainstreaming continues to be a challenge within SOEs in South Africa, as disparities between males and females persist in executive roles and certain technical fields (Balbuena, 2014; Ramparsad, 2021). The Portfolio Committee on Trade, Industry and Competition (2021) also highlights the lack of women’s empowerment in state-owned enterprises (SOEs), particularly noting the shortage of women with necessary technical skills. These disparities highlight possible gender differences among Generation X employees within the sector, which can be manifested in variations in techno-uncertainty and counterproductive work behaviors. Despite all the above evidence, the understanding of whether techno-uncertainty affects counterproductive behaviors remains unexplored in theory. Previous studies (Gugercin, 2020; Kumar, 2024; Shin and Shin, 2024) on counterproductive work behavior have explained it using numerous techno-induced stressors in a single study. However, research specific to only one techno-induced stressor among Generation X employees in SOEs, particularly in the South African context, is scarce. This creates a theoretical gap, that is, a lack of frameworks that: (1) explain the relationship between techno-uncertainty and organizational counterproductive work behavior, (2) consider gender differences in times of adaptability to technology, and (3) account for gender as a moderating variable in these relationships.

Therefore, to close this gap, the current study investigates the moderating role of gender in the relationship between techno-uncertainty and counterproductive work behavior directed at the organization (CWBO) among Generation X employees in a selected SOE in South Africa. Through the integration of strain-related models, the study examines how a technologically induced stressor can lead to negative outcomes that contradict organizational norms, and how gender may influence this relationship. In this regard, the study contributes to theory by extending the known models of counterproductive work behavior in a relationship that considers the role of technology as an antecedent, among Generation X employees within the African context. Including gender in the analysis also extends the applicability of role theory in different organizational and cultural contexts, as well as contributes to the literature on gendered occupational stress.

## 2 Literature review

### 2.1 Theoretical framework

The study is premised on strain-based models, specifically the Job Demands-Resources (JD-R) model (Bakker and Demerouti, 2007; Demerouti et al., 2001) and General The JD_R model states that in the work environment, irrespective of the type of job or occupation, when job demands are high and the job resources to deal with them are limited and unavailable, job strain develops. The General Strain Theory also posits that when a person experiences strain, they tend to develop negative emotions, such as anger and frustration. These two theories help explain the relationship between techuncertainty and counterproductive work behavior among Generation X employees by postulating that the introduction of technology in the workplace makes them feel unable to cope with work demands, resulting in feelings of strain. Feeling unable to cope means they experience limitations in personal resources. The limitations and strains experienced can motivate employees to engage in deviant behavior, such as absenteeism.

### 2.2 Techno-uncertainty

Techno-uncertainty is a dimension of technostress. It represents the frustration of an end user as a result of the fast changes and upgrades of information and communication technologies utilized in their workplace (Tarafdar et al., 2010). Generation X employees, as technology immigrants, may constantly feel pressure to continually update their technological skills so that they can operate new devices and technological upgrades introduced in their workplaces. The pressure may be worsened in situations where modern technology often develops a fault or experiences constant breakdowns, and the employee lacks the capability or knowledge to deal with it. Durndell and Haag (2002) corroborate this argument by reporting that it is always frustrating when a computer or machine suddenly malfunctions, thereby making it impossible for a person to perform their job. Similarly, other studies Liu et al. (2023) have associated techno-uncertainty with unreliable and high-level technology. A study by Spiess et al. (2021) found that the productivity of older employees is reduced due to techno-uncertainty. A similar study (Baham et al., 2023) showed that digital immigrants (Generation X), compared to Generation Y, experienced stress as a result of cognitive overload in situations where computer-managed communication interruptions were important relative to the task. These studies indicate that as technology evolves in organizations, Generation X employees may continue to experience some cognitive challenges and therefore require closer attention.

### 2.3 Organizational counterproductive work behavior

The workplace is like an ecosystem, where working together, being innovative and creative, and being productive are encouraged. However, the ecosystem can be disrupted by the behaviors of some employees that are termed counterproductive. Counterproductive work behavior (CWBO) has long been studied by various authors (Robinson and Bennett, 1995; Vardi and Wiener, 1996; Gruys and Sackett, 2003). A common theme among these authors is that it entails voluntary actions by employees that violate organizational norms, policies, or practices. The behaviors, such as bullying and theft, can be directed at colleagues or the organization, respectively. The most commonly reported in the literature are those behaviors directed at the organization (CWBO) (Mercado et al., 2018). They range from minor transgressions, such as taking excessive breaks, to major incidents, including sabotage. Their occurrence disrupts organizational reputation, introduces costs, and disrupts productivity (Fox et al., 2001). Current studies on generational differences focus more on work values and turnover intentions (Weerarathne et al., 2023), as well as attitudes and inclinations toward digital adoption (Budhe et al., 2023). Few have mentioned differences in counterproductive work behavior, but only among Generation Z and Y (Jauzaa et al., 2023), but not Generation X.

### 2.4 Techno-uncertainty and organizational counterproductive work behavior

Kot (2022) found positive, weak, and moderate correlations between all technostress creators (overload, invasion, complexity, insecurity, uncertainty) and organizational counterproductive behaviors. Harunavamwe and Ward (2022) also provided evidence of the moderating role of all technostress creators (overload, insecurity, uncertainty, and complexity) between techno-inhibitors and deviant behavior, while Chandra et al. (2019) found that techno-uncertainty is positively associated with employee innovation. In contrast, and affirming the General Strain Theory, Shadbad and Biros (2022) demonstrated that techno-uncertainty and other techno-stress creators, such as techno-complexity, were associated with violating ICT compliance behaviors. These two studies demonstrate that techno-uncertainty may or may not be associated with certain negative employee behavioral outcomes. However, one study that buttressed a positive relationship found that technouncertainty was significantly correlated with deviant behavior (Khedhaouriaa and Cucchi, 2019). Based on this discussion, Generation X employees who encounter techno-uncertainty may engage in counterproductive work behavior within organizations. Thus, it is hypothesized that techno-uncertainty has a significant positive influence on organizational counterproductive work behavior.

### 2.5 Moderating role of gender

Several previous studies have provided evidence on how gender differences moderate the relationship between techno-complexity, techno-uncertainty, and counterproductive work behavior. Similarly, Amin et al. (2024) found that technostress decreases productivity, with a more pronounced negative impact on male educators than females.. Interestingly, some studies corroborate these differences, particularly in terms of coping and problem-solving strategies employed by males and females when faced with technostress. For instance, et al. (2020) found differences between males and females in terms of problem-solving strategies, with men taking this approach more than women when faced with psychologically and technically stressful situations. The authors also found differences in risk-taking and technological adaptability between males and females, with males adapting quickly than females. Although none of these studies were conducted among Generation X employees, the observed differences can all be understood within the Social Role Theory (Eagly, 1987), which explains how gender differences in behavior arise from the division of labor and resulting social roles within a society. We therefore propose that gender moderates the relationship between techno-uncertainty and organizational counterproductive work behavior.

**FIGURE 1:**
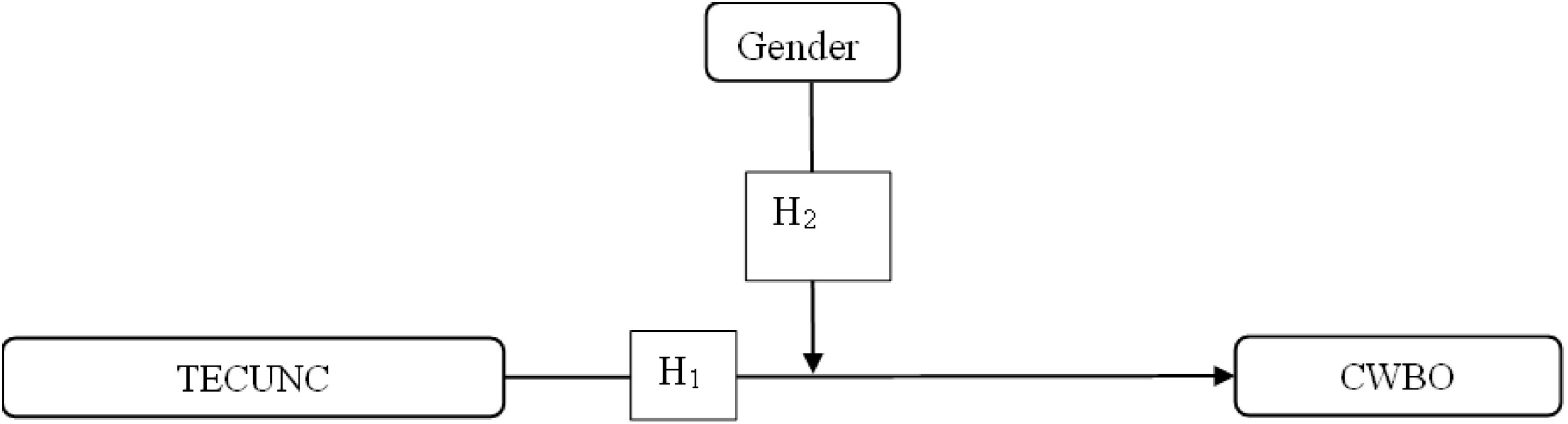
Conceptual framework. TECUNC, techno-uncertainty; CWBO, counterproductive work behavior directed at the organization.

Based on the above literature, the conceptual model (Figure 1) and corresponding hypotheses are presented as follows:

*H1*: Techno-uncertainty (TECUNC) has a significant positive influence on organizational counterproductive work behavior.

*H2*: Gender moderates the relationship between Techno-uncertainty (TECUNC) and organizational counterproductive work behavior (CWBO*).*

## 3 Materials and methods

### 3.1 Study design and sampling

From adopting a positivist research philosophy, which entailed hypotheses formulation and statistical data analysis (Sekaran and Bougie, 2016), data were collected quantitatively. An ex post facto correlational design was adopted because it did not involve the manipulation of any variables (Sharma, 2019). Permanently employed Generation X employees (1965–1980) of one of the SOEs in South Africa were the target population. A non-probability convenience sampling was used to select the sample. The final sample was 230.

### 3.2 Measurement of variables

Organizational counterproductive work behavior was measured using eight items from Aquino et al. (1999) Deviant Behavior Scale. Examples of items are, “Intentionally arrived late at work,” “Made unauthorized use of organization property.” The items were measured on a 4-point Likert Scale ranging from (1) strongly disagree to (4) strongly agree. The instrument developed by Tarafdar et al. (2020) was used to measure techno-uncertainty. The scale consists of five items measured on a 4-point Likert Scale ranging from (1) strongly disagree to (4) strongly agree. Examples of itesm are, “There are always new developments in the technologies we use in our organization” 0.174. The use of a 4-point Likert scale was to ensure that respondents give their opinions relating to their behavior regarding these variables without sitting on the fence.

### 3.3 Test of reliability and validity of constructs

The validation of the formulated hypotheses involved analyzing the data using SmartPLS 4.0 software. Particular attention was given to scrutinizing internal consistency and convergent validity, the latter of which was evaluated utilizing both the Average Variance Extracted (AVE) and factor loadings. Concurrently, internal consistency reliability underwent assessment utilizing metrics such as Cronbach’s Alpha (α) and Composite Reliability (CR). The outer loadings, inclusive of CA, CR, AVE, and factor loadings, were calculated to evaluate the internal consistency across the constructs and are presented in Table 1. Only items that had outer loadings exceeding the recommended threshold were retained.

**TABLE 1:** Reliability and validity results.

Construct	Items	Outer loadings	CA	CR	AVE
TECUNC	TECUNC1	0.867	0.873	0.910	0.717
	TECUNC2	0.846			
	TECUNC3	0.863			
	TECUNC4	0.810			
CWBO	CWBO2	0.750	0.700	0.811	0.589
	CWBO3	0.750			
	CWBO6	0.802			

TECUNC, techno-uncertainty; CWBO, counterproductive work behavior directed at the organization; CA Cronbach alpha; AVE, average variance extracted; CR, composite reliability.

The reliability of the measurement constructs is underscored by the CR and AVE values of items surpassing the recommended thresholds of 0.7 and 0.5, respectively (Hair et al., 2014). Moreover, all CA values falling within the range of 0.700–0.873 signify a high degree of internal consistency. Convergent validity is substantiated by outer loadings exceeding 0.50 and AVE values exceeding 0.50, according to Hair et al. (2014) guidelines. Discriminant validity is evaluated using the Fornell-Larcker criterion, with the findings detailed in Table 2 for further scrutiny and interpretation.

**TABLE 2:** Fronell-Larcker criterion results.

Variables	CWBO	TECUNC
CWBO	**0.767**	
TECUNC	0.190	**0.847**

The number in bold is the square root of AVE. TECUNC, techno-uncertainty; CWBO, organizational counterproductive work behavior.

Discriminant validity is confirmed when the square root of the Average Variance Extracted (AVE) values for each factor (diagonal elements) exceeds the correlation coefficients between factors. To assess multicollinearity, variance inflation factors (VIFs) were examined for all latent variables in the model, with the corresponding values provided in Table 3.

**TABLE 3:** Full collinearity statistics (VIF) results.

Items	CWBO3	CWBO4	CWBO6	TECUNC1	TECUNC2	TECUNC3	TECUNC4
VIF	1.274	1.655	1.378	1.883	2.671	2.640	1.919

TECUNC, techno-uncertainty; CWBO, organizational counterproductive work behavior.

The absence of VIF values surpassing the threshold of 3.3 in the table indicates that multicollinearity among the constructs is not a concern. Before testing the proposed hypotheses, the model underwent thorough examination to ensure it satisfied all model fit indices. R2 and Q2, pivotal in evaluating model fit quality, were assessed, with Penalver et al. (2018) emphasizing the importance of values above zero. Various goodness-of-fit results generated by SmartPLS 4.0 are outlined in Table 4 for reference and further analysis.

**TABLE 4:** Goodness of fit results.

Endogenous latent variable	*R* ^2^	Q^2^	SRMR	NFI
CWBO	0.089	0.057	0.077	0.909

CWBO, organizational counterproductive work behavior; SRMR, standardized root mean square residual; NFI, normed fit index.

The R2 and Q2 values, surpassing zero, indicate predictive relevance for each dependent construct within the path model, aligning with Hair et al. (2016) recommendations. This underscores the overall predictive relevance of the model. Additionally, with a Standardized Root Mean Squared Residual (SRMR) value of 0.077, which is below the 0.08 threshold, the fitted model is considered acceptable. Furthermore, the Normed Fit Index (NFI) value of 0.909, exceeding the recommended threshold of 0.90, further bolsters the model’s acceptability.

## 4 Results

### 4.1 Demographic analysis

Data that was collected revealed 99 (43%) and 131(57%) females. The education data revealed that lower than Grade 12 7(3%), Grade 12(23%), Post-matric certificate 35(15.2%), Diploma/degree 88(38.3%), and Postgraduate 47(20.4%), respectively. In terms of years of work experience, data revealed < 5 years 59(25.7%), 50–10 years 71(30.9%), 11–15 years (30%), and above 16 years 13 (30.5%), respectively. For years in the current organization, the data showed < 5 years 86(37.4%), 5–10 years 65(28.3%), 11–15 years 59(25.7%), and above 16 years 20(8.7%), respectively.

### 4.2 Structural model

To investigate the correlations among the measurement elements depicted in the hypotheses, analysis was performed using SmartPLS software, using the partial least squares (PLS) approach. Table 5 presents a comprehensive summary of the PLS results for the structural model, providing valuable insights into the hypothesized relationships.

**TABLE 5:** Structural model’s PLS results.

		Coefficient			
Hypothesis	Relationship	Std beta	T	*P*-values	Decision
H1	TECUNC-> CWBO	0.174	2.763	0.006	Supported
H2	TECUNC-> Gender-> CWBO	–0.187	2.603	0.009	Supported

TECUNC, techno-uncertainty; CWBO, organizational counterproductive work behavior.

**FIGURE 2:**
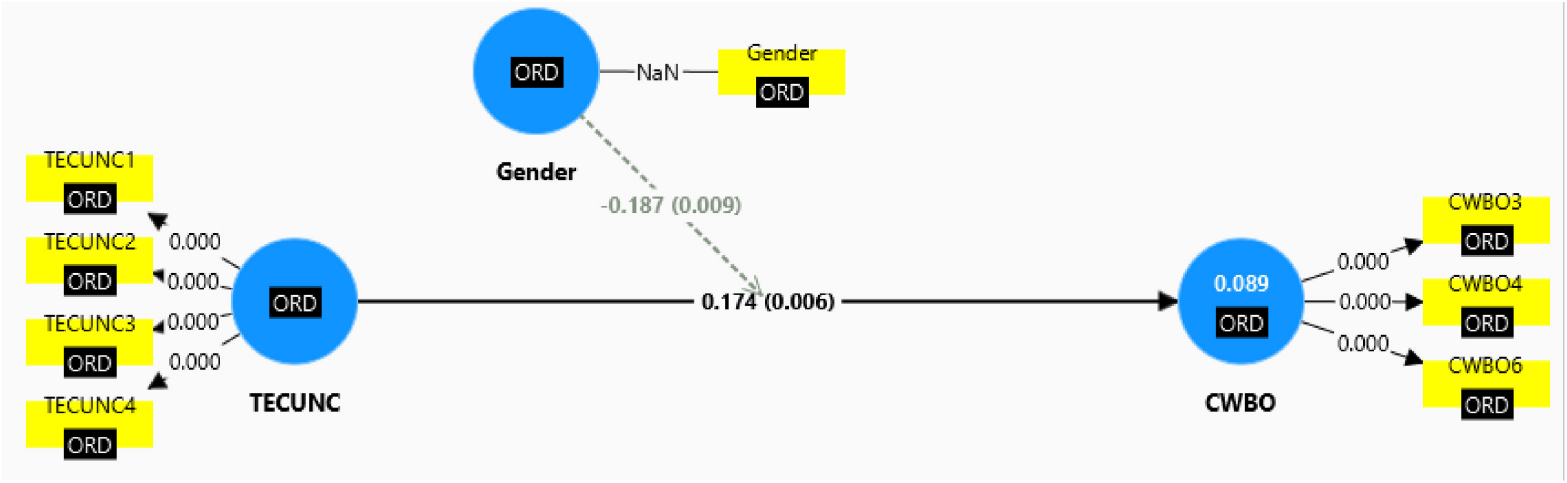
SEM with coefficients.

The insights gleaned from Table 5 are highly compelling. TECUNC exhibits a notable positive effect on CWBO (β = 0.174, *t* = 2.763, *p* = 0.006), indicating an increase in organizational counterproductive work behavior (CWBO) with heightened levels of techno-uncertainty. The corresponding f^2^ value of 0.068 suggests a small effect size based on Cohen’s (1988) guidelines (0.02 = small, 0.15 = medium, 0.35 = large), affirming that while the impact is statistically significant, it is modest in magnitude. Additionally, the analysis reveals that gender significantly moderates the relationship between TECUNC and CWBO (β = –0.187, *t* = 2.603, *p* = 0.009), with an associated *f*^2^-value of 0.036, indicating a small but meaningful effect. These findings provide robust support for Hypotheses 1 and 2, confirming the expected relationships. Figure 2 briefly summarizes the fitted model, capturing coefficients and factor loadings to enhance clarity and facilitate interpretation.

As illustrated in Figure 2, the combined influence of TECUNC and gender explains approximately 8.9% of the total variability observed in organizational counterproductive work behavior (CWBO). This proportion of explained variability signifies a relatively modest portion being captured by the model.

### 4.3 Slope analysis

**FIGURE 3:**
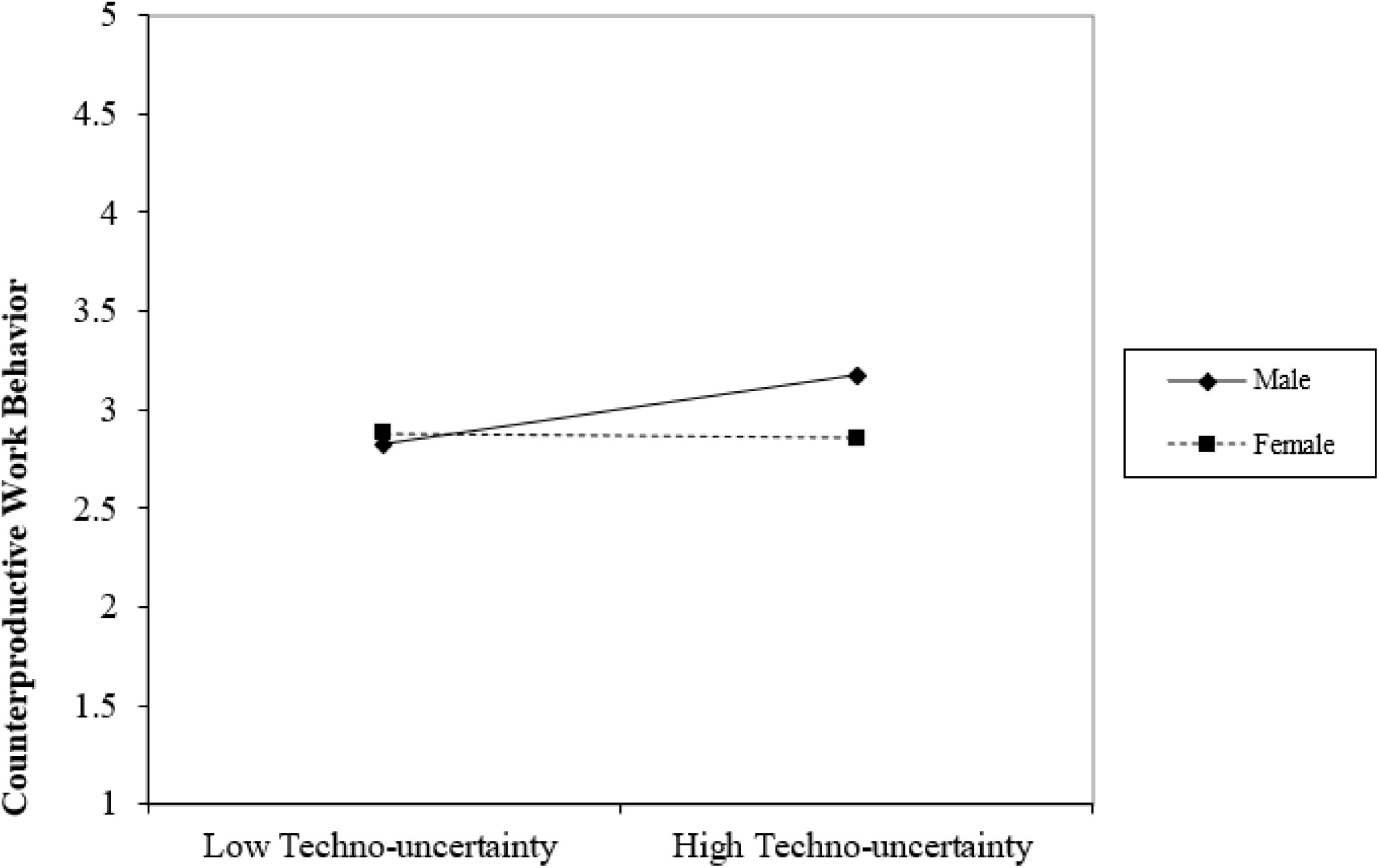
Gender moderation effect.

Considering the substantial impact of gender moderation on the correlation between techno-uncertainty (TECUNC) and organizational counterproductive work behavior (CWBO), a slope analysis was conducted to determine the gender category with the more pronounced influence. The findings of this analysis are depicted in Figure 3.

The results highlight a significant moderate influence of gender on the relationship between techno-uncertainty (TECUNC) and organizational counterproductive work behavior (CWBO). The graph displays a slightly steeper and more positive slope for males compared to females. This indicates a moderate impact of gender on organizational counterproductive work behavior (CWBO) among males compared to females.

## 5 Discussion

### 5.1 Relationship between techno-uncertainty and organizational counterproductive work behavior

It has been argued that Generation X employees would experience techno-uncertainty, and this would lead to their engaging in organizational counterproductive work behaviors. As revealed, it was established that techno-uncertainty has a significant positive impact on organizational counterproductive work behavior directed at the organization. Although the practical effect size is small, this finding confirms the General Strain Theory (Agnew, 1992), which posits that unpleasant experiences lead to frustration and anger. This implies that Generation X employees in SOEs in South Africa are faced with a situation where they constantly feel insecure because constant changes and improvements in technology result in their knowledge and skills becoming obsolete very quickly. Additionally, for Generation X employees, factors such as extended working hours, transferring work outside the workplace, and increased competition from employees who are more skilled in technology may all impact their cognitive and physical health. As a result, they may become frustrated to the extent that they engage in organizational counterproductive work behaviors, such as sabotage, abuse, and theft. Cognisant of the specific behaviors delineated in the current study, the findings support Kot (2022), who found that all technostress creators, including techno-uncertainty, had moderate correlations with counterproductive behavior. However, the study did not split between the two dimensions of the 1. counterproductive work behavior construct. Similarly, Kim and Lee (2020) found that techno-uncertainty is associated with counterproductive work behavior and innovation resistance, while Shadbad and Biros (2022) also showed that techno-uncertainty is positively related to violating ICT compliance behavior. In both studies, however, the samples were not categorically defined as in this study. Notwithstanding this shortcoming and, in tandem with the General Strain Theory (Agnew, 2006), the studies explain the possible strain experienced by Generation X employees when exposed to technology and the subsequent negative behaviors.

In terms of the moderation role of gender, it was found that gender moderates the relationship between techno-uncertainty and organizational counterproductive work behavior directed at the organization. Although the effect size of the moderation is small, the finding meaningfully implies different reactions to technology among Generation X male and female employees, with males experiencing slightly higher techno-uncertainty levels and subsequently exhibiting counterproductive work behaviors directed at the organization. The finding corroborates previous studies by Stich et al. (2017), who found that in the work context, when faced with elevated levels of techno-complexity and uncertainty, females tend to engage in deviant behaviors less than half the time compared to their male counterparts. Similarly, a recent study by Alam et al. (2021) demonstrated a significant difference between female-led and male-led SMEs regarding their perceptions of digital transformation. The current finding also supports the Role Congruity Theory (Eagly and Karau, 2002), which associates societal roles for males and females with different reactions to technological developments. Females may face intense pressure and decide to internalize their frustration, while males may feel threatened by uncertainty and respond with disruptive and harmful behaviors within the organization.

### 5.2 Limitations and suggestions for future research

The study was limited to one SOE in South Africa. While this focus provides in-depth insights into this particular population group, the results may not be generalized to similar organizations in the country or to other sectors and cultural contexts. However, the moderation role of gender found means that future studies may have to replicate and expand upon these results, examining the generalizability of these gender differences across different cultural and organizational settings. The design was such that data was collected at a single point in time and did not take into consideration when the technology was introduced to the sample under study, and how long it had been exposed to it. Future research may have to consider these variations and use a longitudinal approach. The use of convenience sampling may have introduced selection biases, resulting in a sample that does not accurately represent the larger population. Using self-report measures could also have introduced common method bias; therefore, some items were excluded from the final analysis. Future studies may need to avoid such measures and adopt alternative indirect methods of measuring counterproductive work behavior, such as soliciting opinions from supervisors. The other demographic factors, such as age, education level, and years of experience, were not controlled in the study. Future studies may consider a model that incorporates these. The fact that the survey was done in English in a country with 11 official languages may have compromised the results. Future researchers may have to be language-sensitive when doing similar studies. Despite these limitations, the role gender can play in minimizing counterproductive work behavior is clearly shown.

### 5.3 Recommendations for human resource management interventions

The prevalence of organizational counterproductive work behavior suggests valuable insights for Human Resource Management (HRM) interventions in the context of digital and technological adoption by organizations. The interventions can be tailor-made to focus on the dynamics and relationships found in the current research. Therefore, to reduce cases of organizational counterproductive work behaviors, HRM departments need to constantly train Generation X employees, especially when introducing modern technology, through resilience workshops. The goal is to minimize the risk of techno-uncertainty. In addition, human resource managers and supervisors could conduct regular surveys on the impact of gender, attitudes, and competence levels, which may assist organizations in understanding the levels of technology that employees are comfortable with. This has ramifications for the environment-person-job fit decisions. The revision of current policies and codes of conduct by HRM departments to incorporate organizational counterproductive work behaviors associated with digital technology is also critical. The findings on the moderating role of gender highlight the need for HRM interventions that are gender-responsive, addressing the differences between male and female employees across all generational cohorts.

## 6 Conclusion

The study confirms that techno-uncertainty has a positive influence on organizational counterproductive work behavior directed at the organization among Generation X employees. This finding highlights a facet of counterproductive work behavior that is vulnerable to technological disruptions among Generation X employees in a specific organizational and cultural context. The role of gender as a moderating variable in this study also establishes it as a solid boundary condition, rather than a mere control variable, which has the potential to advance models of technostress beyond main effects. The study, therefore, suggests the introduction of gender-sensitive HRM interventions in organizational policies and strategies that are meant to address the impact of digital and technological advancements on different generational employees. Notwithstanding the limitations of the study that may impact the generalizability of the present findings, the study lays the foundation for future studies aimed at incorporating numerous organizations and employees from diverse cultural settings.

## Data Availability

The raw data supporting the conclusions of this article will be made available by the authors, without undue reservation.
